# Nanoparticle Delivered Human Biliverdin Reductase-Based Peptide Increases Glucose Uptake by Activating IRK/Akt/GSK3 Axis: The Peptide Is Effective in the Cell and Wild-Type and Diabetic Ob/Ob Mice

**DOI:** 10.1155/2016/4712053

**Published:** 2016-05-17

**Authors:** Peter E. M. Gibbs, Tihomir Miralem, Nicole Lerner-Marmarosh, Mahin D. Maines

**Affiliations:** Department of Biophysics and Biochemistry, University of Rochester School of Medicine and Dentistry, Rochester, NY 14642, USA

## Abstract

Insulin's stimulation of glucose uptake by binding to the IRK extracellular domain is compromised in diabetes. We have recently described an unprecedented approach to stimulating glucose uptake. KYCCSRK (P2) peptide, corresponding to the C-terminal segment of hBVR, was effective in binding to and inducing conformational change in the IRK intracellular kinase domain. Although myristoylated P2, made of L-amino acids, was effective in cell culture, its use for animal studies was unsuitable. We developed a peptidase-resistant formulation of the peptide that was efficient in both mice and cell culture systems. The peptide was constructed of D-amino acids, in reverse order, and blocked at both termini. Delivery of the encapsulated peptide to HepG2 and HSKM cells was confirmed by its prolonged effect on stimulation of glucose uptake (>6 h). The peptide improved glucose clearance in both wild-type and Ob/Ob mice; it lowered blood glucose levels and suppressed glucose-stimulated insulin secretion. IRK activity was stimulated in the liver of treated mice and in cultured cells. The peptide potentiated function of IRK's downstream effector, Akt-GSK3-(α, β) axis. Thus, P2-based approach can be used for improving glucose uptake by cells. Also, it allows for screening peptides* in vitro* and in animal models for treatment of diabetes.

## 1. Introduction

The defect leading to type 1 diabetes is autoimmune ablation of the pancreatic *β*-cells, including those arising from infection, causing severely impaired glucose uptake from the circulation [1, 2]. In type 2 diabetes, the insulin signaling cascade is impaired in the insulin-responsive tissues: skeletal muscle, adipose tissue, and liver [3]. The action of insulin as a metabolic regulator and a growth factor is protein tyrosine kinase- (PTK-) dependent and is an essential step in the initiation of signaling cascade initiated by its cell surface receptor. The insulin receptor is a heterotetramer of two *α*- and two *β*-subunits [4, 5]. The *α*-subunits are entirely extracellular and contain the ligand-binding site. The *β*-subunits are composed of three domains: extracellular, which is disulfide bonded to the C-terminal region of the *α*-subunits, transmembrane, and cytosolic. The cytosolic domain including a tyrosine kinase domain (IRK) contains three tyrosine residues, Y^1158,1162,1163^, that are autophosphorylated upon ligand binding to the *α*-subunits. Autophosphorylation stimulates a change in conformation of the activation loop that is necessary for binding and tyrosine phosphorylation of proteins containing YMXM sequence [6], including IRS1, IRS2, PI3K, and BVR, and allows assembly of multiprotein signaling complexes [7–12]. Defective insulin signal transduction is associated with molecular defects in the signaling pathway, the evaluation of which has largely focused on major nodes in the pathways, the insulin receptor itself, insulin receptor substrates 1 and 2, PI3K, Akt/PKB, atypical PKCs (aPKCs, i.e., *ζ* or *λ*), and MAPKs [3].

Activation of IRK stimulates the Akt/GSK axis, which is vital for glucose uptake and metabolism [18, 19]. Activated IRK phosphorylates the adapter molecules IRS1 and IRS2, which in turn recruit other proteins to the complex including PI3K. Activation of PI3K results in synthesis of phosphatidylinositol-3-phosphates, which act as membrane anchors for pleckstrin homology domain-containing proteins, such as PKCs, PDK1, and Akts(1–3). Three Akt isoforms have been characterized [20, 21]; Akt1 is the most intensely studied isoform. Unlike Akt1 or Akt2, Akt3, which is highly expressed in testis and brain, appears to play no role in glucose homeostasis [22, 23]. The catalytic domain of all Akt kinases has a threonine residue in the activation loop, T^308^, that is phosphorylated by PDK1 after both proteins have been recruited to the membrane. A second phosphorylation, at S^473^ in the hydrophobic loop, leads to maximal activity [15, 24]. The mechanism of serine phosphorylation has been attributed to autophosphorylation and to several other kinases [25, 26]. Threonine^450^ in the turn motif of the C-terminal regulatory domain is also a phosphorylation target; modification of this residue, however, is not required for full activity. Activated Akt in turn regulates a wide variety of cellular functions, including that of one of its substrates, glucose synthase kinase-3 (GSK3). The GSK3*α* and GSK3*β* isoforms are inactivated after phosphorylation by Akt; inactivation of GSK3 stimulates glucose uptake and also allows activation of glycogen synthase, and hence synthesis of glycogen [27, 28].

Human (h)BVR is a 296-residue soluble protein that was initially described as being the sole cellular source of bilirubin, a most potent intracellular quencher of free radicals, including both reactive oxygen and reactive nitrogen species derived from O_2_ and NO, respectively [29–34]. However, hBVR is also a bZip (basic zipper) transcription factor for regulation of stress response genes, including ATF2/CREB [35, 36] and a Ser/Thr/Tyr kinase that is activated by IRK [12, 37]. hBVR translocates, depending on the stimulus, to and from the nucleus, cytoplasm, or cell membrane [16, 38–40] and in so doing functions as a scaffold and as an intracellular carrier protein [16, 35, 36, 39, 41, 42]. The structural features of the protein are of relevance to its many functions [43–45]; the N-terminal domain includes the active site and residues involved in ATP/NADPH binding, whereas the C-terminal domain includes a large six-stranded *β*-sheet followed by an *α*-helix that forms an ideal surface for protein : protein interaction.

Insulin was shown to regulate components of heme degradation pathway. It induced HO-1 expression through IRS1/PI3K/Akt2 pathway [46]. Also, hBVR was shown to be a substrate for the insulin receptor kinase [12], which phosphorylates three tyrosine residues in the protein : protein interactive domain* in vitro*. One of these, Y^198^, is in a canonical IRK substrate motif, YMKM, while Y^228^ in the YLSF motif that meets YΦSΦ criteria for an IRK target [47] and Y^291^ is in the C-terminal helix of hBVR (aa 271–296) [12]. We have systematically dissected hBVR functions using synthetic peptides based on its primary sequence and tested the peptides* in vitro* and in cell culture systems; one such fragment corresponding to its C-terminal 7 residues, K^291^YCCRSK (hereinafter P2), is an activator of IRK, by means of a novel intracellular interaction with the kinase and stimulator of glucose uptake [48]. The peptide was shown to increase IRK *V*_max_  , without changing *K*_*m*_ of the kinase. It stimulated glucose uptake in 4 cell lines tested so far. Change in fluorescence emission spectra of IRK domain (aa 988–1263), with fluorophore coupled to cysteines, C^1056^, C^1138^, or C^1234^ in the presence of KYCCSRK, indicated that the peptide bound to and changed IRK conformation. Binding of the sequence to IRK was substantiated by finding that KYCCSRK sequence in intact hBVR was necessary for the hBVR/IRK cross-activation [48].

We have demonstrated that P2 stimulates glucose uptake in several cell types, including cells derived from liver (HepG2), kidney (HEK), pulmonary artery smooth muscle (PASM), and skeletal muscle myoblasts, using an N-myristoylated form of the peptide synthesized using the naturally occurring L-enantiomeric amino acids [48]. This form was membrane permeable and effective for a brief period in the cultured cells. For a number of reasons, this composition is unsuitable for use as a therapeutic agent; small molecules (MW < 5 kDa) are rapidly depleted in the circulation and excreted via the kidneys, and moreover the L-amino acid peptides are highly susceptible to proteolytic degradation [49, 50]. Accordingly, we generated a nanoparticle formulation of P2 containing the peptidase-resistant D-enantiomeric form of P2. This proved to be more effective in increasing glucose uptake by cultured cells than myristoylated P2 and decreased glucose level in mice. We measured IRK activity and glucose uptake and examined whether modulation of IRK activity and glucose uptake involves Akt/GSK pathway. We found the construct effective in ameliorating hyperglycemia and in stimulating the IRK/Akt/GSK axis. The mechanism was demonstrated to be that predicted for activation of IRK.

## 2. Materials and Methods

### 2.1. Materials

Peptides with all D-amino acids—Ac-KRSCCYK-NH_2_ and Ac-YKCKCRS-NH_2_—and myristoylated peptides were synthesized by EZ Biolabs (Carmel, IN). Heparin (porcine) was obtained from Thermo-Fisher, protamine was from EMD-Calbiochem, 1,2-dioleoyl-3-trimethylammonium-propane chloride (DOTAP) was from Avanti Polar Lipids (Alabaster, AL), and cholesterol (>99% purity) was from Sigma-Aldrich. Insulin receptor *β*-subunit (IRK), IGF receptor *β*-subunit (IGFR kinase), IRS, and IRS Y^608^ peptide substrate were from Enzo Life Sciences (Farmingdale, NY), [*γ*-^32^P]-ATP was from Perkin-Elmer, and [1-^3^H]-2-deoxyglucose was from Amersham. Antibodies were obtained from Cell Signaling (Beverly, MA).

#### 2.1.1. Peptides Used in the Study

Myristoylated peptides were synthesized with L-amino acids. The peptides were as follows: P2, myr-KYCCSRK; P2sc, myr-SRCKCKY; control, peptide-A myr-KKRILHC; peptide-B myr-KRNRYLSF. Two peptides contained only D-amino acids: DP2 Ac-RSCCYK-NH_2_ and D control Ac-YKCKCRS-NH_2_.

#### 2.1.2. Preparation of Nanoparticles

Nanoparticle assembly was based on a previously described method [51]. Membrane lipids were prepared from a 2 mL solution of 18 mM DOTAP and 18 mM cholesterol in 3 : 1 chloroform : methanol. The solvent was evaporated under constant airflow, followed by vacuum drying overnight. The lipids were then slowly suspended in 2 mL PBS, and the suspension was stored at −20°. Unilamellar membranes were prepared by extrusion of the lipid stock through a 100 nm filter and were used within one week.

Heparin (2.5 mg/mL) and protamine (2.0 mg/mL) stocks were prepared in 20 mM Hepes, pH 7.5, and filter sterilized (0.2 *μ*m). Peptides were dissolved in PBS at 2 mM. In initial experiments, peptide and heparin were combined (1 : 1 v : v), after which increasing amounts of protamine stock were added, and the volume was adjusted to yield a final peptide concentration of 200 nM. The particle sizes were determined by dynamic light scattering (DLS) using a Dynapro II Plate Reader (Wyatt Instruments, Goleta, CA). The particle core formulation for subsequent experiments was based on these data: cores consisted of 250 *μ*g/mL heparin, 200 nM peptide, and 75 *μ*g/mL protamine. The core particles were titrated with membranes (hydrodynamic radius of ~100 nm by DLS), and the size was again assessed by DLS.

#### 2.1.3. Cell Culture and Transfection

Cultures of HEK293 were maintained in DMEM with 10% FBS. Cells were starved overnight in medium with 0.1% FBS. When used, insulin was added to serum-starved cultures at 100 nM. Human skeletal muscle cells were maintained in 1 : 1 DMEM : F12 medium containing 15% FBS. One day prior to glucose uptake analysis, the medium was changed to low glucose (5 mM) DMEM with 10% FBS.

#### 2.1.4. Glucose Uptake in Cells

Glucose uptake was assessed using 2-deoxy-1-[^3^H] glucose as described previously [12], with modification. Cells in 24-well plates were grown to near confluence and starved in glucose-free DMEM (Invitrogen) containing 1 mg/mL BSA for 2 h. Peptides (20 *μ*M) were added and incubation continued for the times indicated in legends of Figure 2, followed by treatment with 100 nM insulin for 15 min. Deoxyglucose (0.2 mM, containing 1 *μ*Ci/mL [^3^H]-deoxyglucose) was added for 15 min. Cells were washed 3 times with cold PBS and solubilized in 150 *μ*L 50 mM NaOH. Radioactivity was measured and normalized to protein concentration. Assessments were made in triplicate and experiments were repeated three times.

#### 2.1.5. Akt and GSK3 Activation

HEK cells were incubated in low glucose DMEM with 0.1% FBS, overnight. Cells were treated with 40 *μ*M myristoylated peptide for a total of 15 min; insulin or IGF1, if used, was included for the final 10 min. Cells were harvested and lysed in buffer with triton X100, and whole cell lysates were analyzed for phosphorylated Akt (T^308^, S^473^) or GSK3 (*α*-S^21^, *β*-S^9^) species by Western blotting. Each blot was stripped and probed with anti-Akt1 or anti-GSK3*α* or anti-GSK3*β* antibody, as appropriate, to control for loading.

#### 2.1.6. Kinase Assays

Purified IRK was assayed as previously [12], using 300 *μ*M IRS1 Y^608^ peptide substrate. IRK autophosphorylation was determined as described earlier [12]. hBVR-based peptides (20 *μ*M) were added as activators or inhibitors. In some experiments, the hBVR-based peptides were themselves used as substrates. Incorporation of ^32^P was determined by liquid scintillation counting [12]. For IRK assays in cells or tissue samples, the protein was solubilized in buffer containing triton X100 and DTT and immunoprecipitated with antibody to IR *β*-chain. Immunoprecipitates bound to protein A/G-agarose were washed in lysis buffer, followed by IRK assay buffer, and kinase activity was measured as before [12]. Akt kinase activity was measured in 50 mM HEPES, pH 7.4, 50 mM MgCl_2_, 0.5 mM EGTA, 2 mM DTT, and 100 *μ*M ATP (containing 10 *μ*Ci [*γ* ^32^P]-ATP) with 30 *μ*M Crosstide (GRPRTSSFAEGKK) substrate; incorporated ^32^P was measured as above.

#### 2.1.7. Animal Studies

C57BL/6J and homozygous* Ob/Ob* mutant mice with the same genetic background were obtained from Jackson Laboratories. For glucose tolerance tests, wt mice were deprived of food for 12 h prior to the experiment. Mice were anesthetized with subcutaneous ketamine (150 mg/kg). In some experiments, ketamine-xylazine (80 and 4 mg/kg, resp.) was used, but this was discontinued due to the hyperglycemia induced by xylazine [52]. Blood was obtained from the tail, and the blood glucose was measured using a portable glucometer (Contour, Bayer, Mishawaka, IN) prior to injection. Nanoparticles (1.1 *μ*g/g peptide) were injected intraperitoneally, followed 10 min later by injection with glucose (1.5 mg/g, ip). Blood was collected at intervals after administration of glucose, and the blood glucose was measured; at least two readings were made at each time point. Glucose stimulation of insulin secretion was measured in mice treated with P2- or control nanoparticles. Prior to injection with particles, ~25 *μ*L of blood was collected into tubes containing 2.5 *μ*L 50 mM EDTA in PBS. 10 min after injection of nanoparticles (1.1 *μ*g/g), glucose (0.5 mg/g body weight) was administered ip, after which blood was collected at 2, 5, and 10 min to measure glucose-stimulated insulin secretion [53]; insulin levels in 5 *μ*L whole blood were determined by ELISA (Alpco, Salem, NH).

### 2.2. Statistical Analysis

Besides animal studies, all experiments were performed at least three times. Statistical analysis by one-way ANOVA and nonlinear regression were performed using Prism software (Graphpad, San Diego).

## 3. Results

Although they have advantages over small molecules in that they have a higher affinity/specificity to target and lower toxicity in the intact body, peptides exhibit a short dwell time due to both rapid renal clearance and lack of stability due to protease degradation. Furthermore, peptides have limited access to intracellular space. In order to reduce degradation by serum and tissue peptidases* in vivo*, chemical alterations of peptides are necessary, such as the use of nonnatural D-amino acids, which substantially prevents peptide degradation, as they are poorly recognized by peptidases. The purpose of this study was to develop a means of delivering the biologically active peptide to animals, in a form that would allow it to persist in the organism. The modified peptide was synthesized using D-amino acids with reversed sequence to maintain the secondary structure. Furthermore, both the N- and C-termini were blocked, using acetyl and amido groups, respectively [49, 50]. Because, in general, the size of particles is inversely proportional to its clearance time from circulation [49, 50], it was also essential that the mode of delivery be modified, to protect the peptide against such rapid loss. To this end, we elected to use particles ~100 nm in diameter, which should persist in the circulation long enough to reach target cells. Lipid-encapsulated particles that are relatively homogeneous in size and carry a net positive charge should facilitate uptake by cells.

### 3.1. Characterization of Nanoparticles

**Figure 1:**
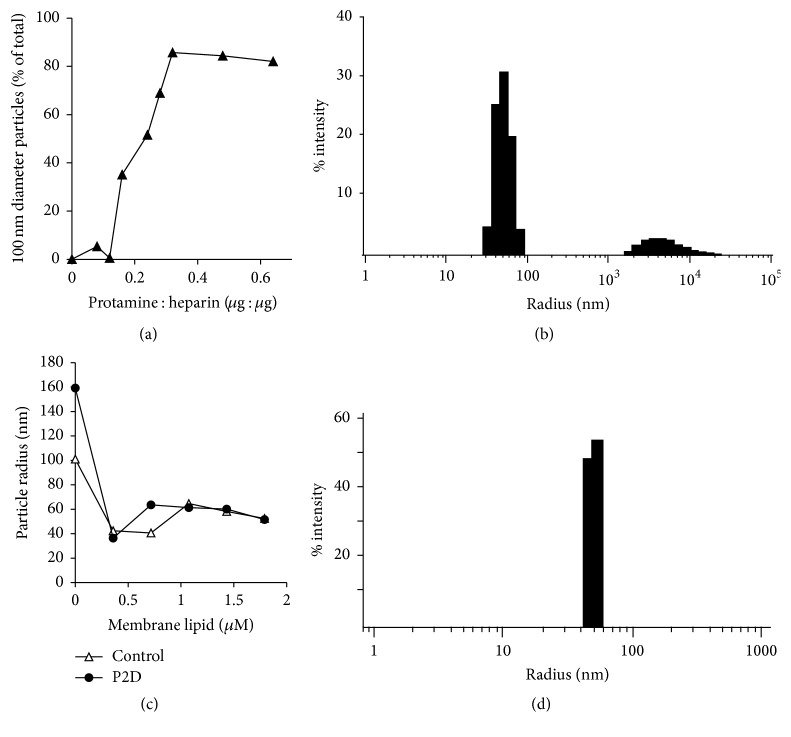
Construction of nanoparticles. (a) Titration of heparin-peptide complex with protamine. Protamine samples (60 *μ*L) containing 15 *μ*g heparin and 200 nM peptide in PBS were tested for the particle hydrodynamic radius by dynamic light scattering (DLS). The proportion of particles with a radius of ~100 nm is plotted. (b) Representative DLS profile; peptide-heparin-protamine core. Representative DLS profile of particles containing 0.3 : 1 protamine : heparin (w : w) together with 200 *μ*M P2 (KYCCSRK). (c) Titration of core complex with membrane lipids. Core particles were prepared as in (b), and increasing amounts of membrane particles (Section 2) were added prior to measurement of hydrodynamic radius by DLS. P2D P2 peptide built of D-amino acids. (d) Representative DLS profile, fully assembled nanoparticles. Representative DLS profile of particles with core structures prepared as in (b), after addition of 0.4 mM membrane lipid.

The first stage was construction of a suitably sized core containing peptide, heparin, and protamine [51]. Initially, a stock containing heparin and peptide was titrated with protamine, and the resulting material was analyzed by dynamic light scattering (Figure 1(a)). At low protamine : heparin ratios, the particles were generally small and heterogeneous in size. However, at a ratio of 0.3 : 1, approximately 80–90% of the particles had a hydrodynamic radius of approximately 80–100 nm (Figure 1(b)). No further increase in the proportion of particles in this size class was seen at higher concentrations of protamine; in some instances, there was a tendency for larger aggregates to form. These data enabled construction of a standardized core that contained 250 *μ*g/mL heparin, 200 nM peptide, and 75 *μ*g/mL protamine. Unilamellar membranes, containing an equimolar ratio of cholesterol and DOTAP, were prepared by extrusion through a 0.1 *μ*m filter. The resulting membrane particles had a mean hydrodynamic radius of 100 nm, as measured by DLS. Core particles, prepared as above, were then titrated with membranes (Figure 1(c)); the control particles in this experiment contained 250 *μ*g/mL heparin and 90 *μ*g/mL protamine, with no P2. Addition of 0.4 *μ*M membrane to the core particles yielded a homogeneous distribution of particles with a hydrodynamic radius of ~50 nm (Figure 1(d)).

### 3.2. D-Peptide Nanoparticles Potentiate Glucose Uptake

**Figure 2:**
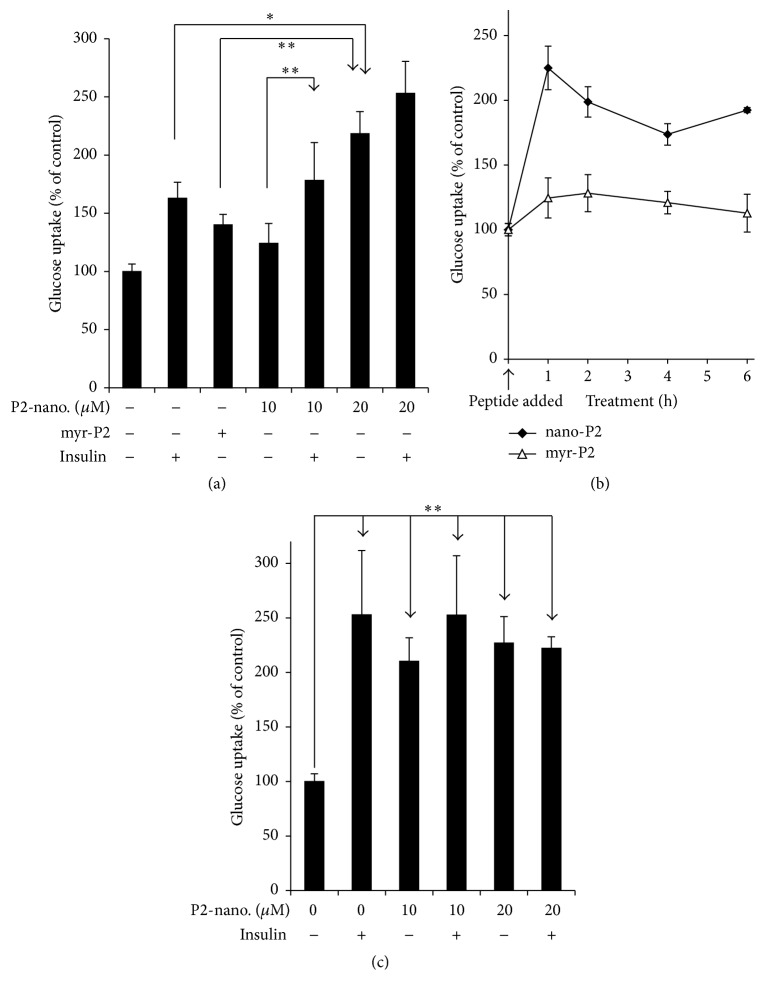
P2-nanoparticles induce glucose uptake in cells. (a) Nanoparticle delivery of peptide P2 (KYCCSRK) to human skeletal muscle (HSKM) cells stimulates glucose uptake. HSKM cells were grown in low glucose, low FBS medium overnight; then media were replaced with PBS containing 1% BSA for 1 h. Cells were pretreated for 2 h with 20 *μ*M myristoylated P2 or with 10 or 20 *μ*M P2-nanoparticles, followed by measuring ^3^H-deoxyglucose uptake over a 15 min interval. Insulin, when used, was added at the same time as ^3^H-deoxyglucose. Each value is the mean ± sem of five samples. ^*∗*^ *P* < 0.05, ^*∗∗*^ *P* < 0.01. (b) Time course of glucose uptake stimulation by P2. HSKM cells were starved overnight as in (a). Medium was changed to glucose-free DMEM with 1% BSA for 1 h prior to treatment with 20 *μ*M myr-P2 or with P2-nanoparticles for the indicated times. Glucose uptake was measured as in (a), with each value being the mean ± sem of five samples. (c) P2-nanoparticles affect glucose uptake in HepG2 cells. HepG2 cells were starved in low glucose DMEM with 0.1% FBS overnight. Treatment with peptide nanoparticles was for 1 h. Glucose uptake was measured as in (a).

P2 is known to stimulate glucose uptake, whether it is delivered as myr-P2 or if it is expressed in cells transfected with a suitable expression plasmid [48]. The nanoparticle delivery system was compared with myristoylated peptide for its effect on glucose uptake by cultured cells. To enable comparison with our earlier study, myr-P2 was administered at a final concentration of 20 *μ*M; the same dose was used for the nanoparticles, and in preliminary experiments a lower dose of 10 *μ*M nanoparticle peptide was also examined. Cells were treated with peptide for 2 h, after which glucose uptake was measured (Figure 2(a)). It was apparent that myr-P2 and insulin stimulated glucose uptake to about the same extent, whereas the nanoparticles were significantly more effective. The lower dose of P2-nanoparticles tested did not effectively increase glucose uptake, although the lower dose combined with insulin was more effective than either low dose peptide or insulin alone (Figure 2(a)). The distinction was largely lost at the higher peptide dose. Bearing in mind that the D-amino acid peptide was designed to be more stable in the intracellular environment, the data cannot distinguish between peptide delivery being more efficient and reduced turnover of peptide in the cells. In a second experiment, cells were treated with 20 *μ*M myr-P2 or P2-nanoparticles for times ranging from 1 to 6 h, after which glucose uptake was measured. It is apparent from Figure 2(b) that the nanoparticle treated cells showed enhanced glucose uptake at all times examined. It is also noted that the initial, albeit lesser, stimulation of glucose uptake by myr-P2 was effectively lost by 6 h, indicating that the myr-P2 is less stable in the cell than the D-form P2-nanoparticles. It is also unclear why the myristoylated peptide showed such poor stimulation of glucose uptake in this experiment. The effect of peptide was not cell-type specific, since HepG2 cells also showed enhanced glucose uptake after treatment with peptide nanoparticles (Figure 2(c)). The nanoparticle treatment time was only 1 h, in contrast to Figure 2(a); however, nanoparticle treatment for 1 h clearly is adequate to manifest the effect of the peptide, as shown in Figure 2(b). Uptake by these cells was stimulated to a greater extent in response to the lower peptide dose than was seen with the skeletal muscle cells in Figure 2(a). In this experiment, however, the cells had been subjected to a longer period of serum deprivation than the skeletal muscle cells, which resulted in a more robust response to insulin; in our hands, the skeletal muscle cells, unlike HepG2 or indeed other cell lines that we have used [48], did not adapt well to being maintained overnight in medium with low (0.1%) FBS and tended to detach from the culture dish.

### 3.3. P2-Nanoparticles Modulate Circulating Glucose Levels* In Vivo*

**Figure 3:**
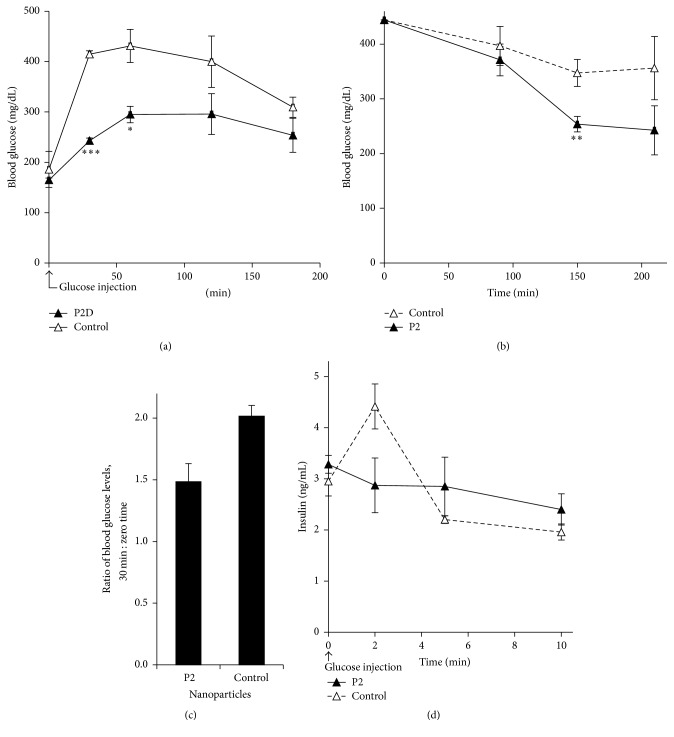
P2-nanoparticles modulate circulating glucose level. (a) Glucose tolerance test in wild-type mice; P2-nanoparticles lower blood glucose level. C57Bl/6J mice were deprived of food overnight. After anesthesia, P2- or control nanoparticles (1.1 *μ*g peptide/g body weight) were injected ip, followed by glucose injection 10 min later (1.5 mg/g body weight). Blood glucose was measured with a portable glucometer at the times shown. The values are mean ± sem for four mice in each group. ^*∗*^ *P* < 0.05, ^*∗∗∗*^ *P* < 0.001. (b) Glucose tolerance test in Ob/Ob mice-P2-nanoparticles lower blood glucose level. Anesthetized mice were injected with P2- or control nanoparticles, and blood glucose was measured starting 30 min later, for the times indicated in the figure. Data points are mean ± sem for three mice in each group ^*∗∗*^ *P* < 0.01. (c) P2-nanoparticles attenuate hyperglycemia induced by ketamine-xylazine anesthesia in fed Ob/Ob mice. Blood was withdrawn for glucose measurement as soon as the mice became unconscious. Second sample was taken at 30 min after nanoparticle injection. Data are presented as the mean ratio of blood glucose at 30 min to that at zero time, ± sem. The difference in mean ratio is significant; *P* < 0.05. (d) Glucose stimulation of insulin secretion is suppressed by P2-nanoparticles in Ob/Ob mice. Ob/Ob mice were injected with P2- or control nanoparticles, followed by glucose injection (0.5 mg/g body weight). Blood was withdrawn at the indicated times, and insulin was measured by ELISA. The data shown are the mean ± sem for three mice in each group.

The experimental data above established that the nanoparticles deliver the active P2 to the cell, leading to increased uptake of glucose, confirming our earlier observations with P2 delivered as a myr-peptide or expressed in the cell. The current delivery system was designed to deliver the peptide to an intact animal and we therefore examined its effect on circulating glucose in normal mice subjected to a high dose of glucose and in obese mice with high circulating glucose, or in response to a stress-induced high blood glucose. First, we examined the effect of nanoparticle-P2 on glucose clearance from the blood, using a glucose tolerance test. It was found that mice injected with P2 (as nanoparticles) 10 min prior to glucose cleared blood glucose much faster than their counterparts injected with a control peptide (Figure 3(a)); the control peptide has the same amino acid composition but an altered sequence (Section 2). As noted in the figure, the P2-injected mice showed significantly lower blood glucose than the controls at 30 and 60 min (*P* < 0.001, *P* < 0.05, resp.). P2 treatment is therefore driving rapid glucose clearance from the circulation.

We next examined the effect of the peptide on glucose uptake from the circulation in Ob/Ob mutant mice, as a model of type 2 diabetes, that had been fed* ad libitum*. In one experiment, the mice were injected with peptide nanoparticles, and blood glucose was monitored starting at 30 min after injection and continuing for a further 3.5 h (Figure 3(b)). The P2 injected mice showed a steady reduction in blood glucose levels over the course of the experiment. Glucose levels in mice injected with control particles also showed a time-dependent decrease, but the rate and magnitude of decrease were lower. It has been reported by Saha et al. [52] that ketamine-xylazine anesthesia induces hyperglycemia in experimental mice. We therefore tested the effect of P2-nanoparticles on this phenomenon. Peptide nanoparticles were injected into Ob/Ob mice when anesthesia was established. Comparison of blood glucose before and 30 min after injection with peptide (Figure 3(c)) revealed that blood glucose in control-injected mice increased by a factor of 2.02 ± 0.09, compared to only a 1.49 ± 0.15-fold increase in the P2-treated animals (*P* = 0.02).

A rapid increase in blood glucose triggers insulin secretion, which in turn activates mechanisms for glucose uptake. We therefore tested whether P2-nanoparticles affected the glucose-stimulated insulin secretion. The following experiment with the obese mice examined the stimulation of insulin secretion in response to glucose in control and P2-treated mice. A modest stimulation of insulin secretion was seen in control animals (Figure 3(d)). However, treatment with P2 10 min prior to injection of glucose prevented glucose stimulation of insulin secretion, most likely because it prevents blood glucose levels from attaining the threshold to stimulate release from pancreatic *β*-cells. Taken together, the data suggest that P2 is an effective stimulator of glucose uptake from the circulation, thereby ameliorating hyperglycemia. It is likely that the peptide is acting independently of insulin; the peptide and insulin both activate the receptor, but by different mechanisms [48].

### 3.4. P2 Activation of IRK

**Figure 4:**
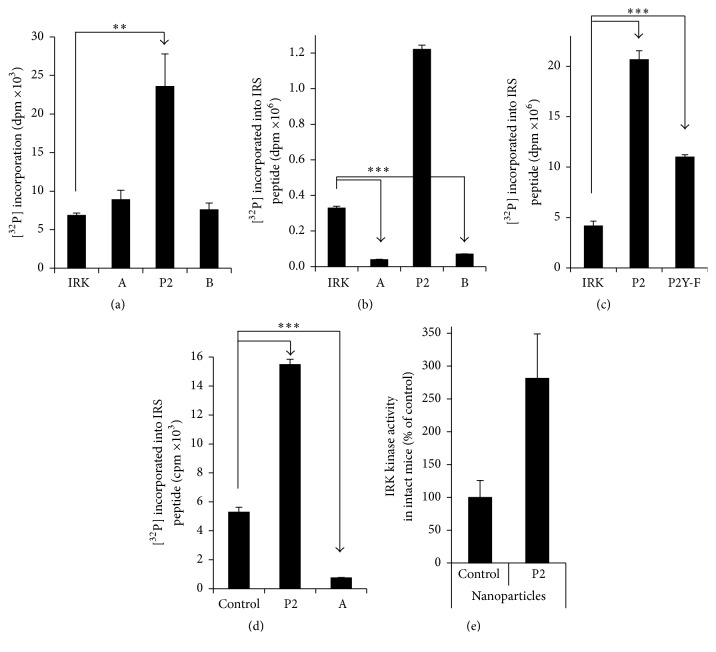
P2 activation of IRK. (a) P2 stimulate IRK autophosphorylation* in vitro*. IRK was incubated* in vitro* with 20 *μ*M of peptide P2 (KYCCSRK), peptide-A (KKRILHC), or peptide-B (KRNRYLSF) and [^32^P]-ATP. The [^32^P] incorporation was quantified as described in Section 2,  ^*∗∗*^ *P* < 0.01. (b) P2 stimulate IRK kinase activity* in vitro*, identification of IRK inhibitory peptide. A similar assay was performed as in (a), except that the IRK peptide substrate was included ^*∗∗∗*^ *P* < 0.001. (c) P2 is both a substrate and activator of IRK kinase activity* in vitro*. Assays using sequence variant (P2Y-F; KFCCSRK) of P2 were performed as in (b) ^*∗∗∗*^ *P* < 0.001. (d) P2 stimulates IRK kinase activity in the cell; peptide-A is an inhibitor. HEK cells were serum-starved overnight and treated with the indicated myristoylated peptides (40 *μ*M) for 15 min. IRK was immunoprecipitated from cell lysates and assayed using the IRS peptide as substrate ^*∗∗∗*^ *P* < 0.001. (e) Activation of liver IRK by nanoparticle P2. C57Bl/6J mice were deprived of food overnight, anesthetized with ketamine, and injected with P2- or control nanoparticles. The mice were sacrificed 30 min later, and the liver was harvested. 100–200 mg liver was homogenized; IRK was assayed in immunoprecipitates as in (d). The data shown are the mean ± sem for four mice under each treatment.

To elucidate the mechanism by which P2-nanoparticles are acting, we sought to confirm and extend our earlier observations that P2 is activating IRK [48]. First, P2 enhances the activity of IRK in an autophosphorylation reaction (Figure 4(a)). It should be noted that IRK is a tyrosine kinase and that P2 contains a tyrosine residue that is phosphorylated by IRK in full-length hBVR [12]; it is therefore possible that the peptide is functioning as a substrate in this reaction. Inclusion of two other BVR-based peptides (peptide-A, KKRILHC and peptide-B, KRNRYLSF) did not lead to increased [^32^P] incorporation in this assay (Figure 4(a)), suggesting specificity to P2 activation. Since increased IRK autophosphorylation results in increased kinase activity, the effect of P2 on IRK kinase activity towards an IRS peptide substrate was tested. As expected, inclusion of P2 led to an approximately fourfold increase in IRK-dependent incorporation of [^32^P] into the peptide substrate when compared to IRK alone (Figure 4(b)). In contrast, other BVR-based peptides (including those used in panel (a)) inhibited the kinase activity. Peptide-B has previously been shown to inhibit insulin-dependent stimulation of glucose uptake [48] and it is probable that it does so by inhibiting cellular IRK. The tyrosine residue in P2 appears to be necessary for complete activation; mutation of the residue to phenylalanine results in a significant decrease in substrate phosphorylation (Figure 4(c)). These observations confirm and extend those made in an earlier study [48].

We then examined IRK activation in cultured cells treated with myristoylated peptides. Cells were lysed 15 min after treatment with peptide and the IR *β*-chain was immunoprecipitated from the lysates. The kinase activity of the immunoprecipitated protein was measured using the IRS peptide substrate. The findings parallel those seen in the* in vitro* assay, in that P2 activated IRK approximately 3-fold in the cell (Figure 4(d)), while peptide-A was a potent inhibitor of the kinase.

To test whether the same activation was seen* in vivo*, C57Bl/6J mice were injected with P2- or control nanoparticles. They were sacrificed 30 min after injection, and the livers were harvested. Liver IRK was isolated from homogenates by immunoprecipitation with antibody against the IR *β*-chain, and kinase activity in the immunoprecipitates was determined. The IRK activity of individual mice is shown in Figure 4(e). It is apparent that there is variation between individual animals. In general, the mice injected with P2-nanoparticles displayed higher IRK activity than the mice injected with control particles, and a two-tailed *t*-test revealed that the difference was statistically significant (P2-nanoparticles, 3.58 ± 0.86 (sem), versus control, 1.27 ± 0.33; *P* < 0.05). It is therefore likely that the increased glucose uptake seen in mice injected with the P2-nanoparticles (Figure 3) is a consequence of increased IRK activity.

### 3.5. P2 Activation of Akt

**Figure 5:**
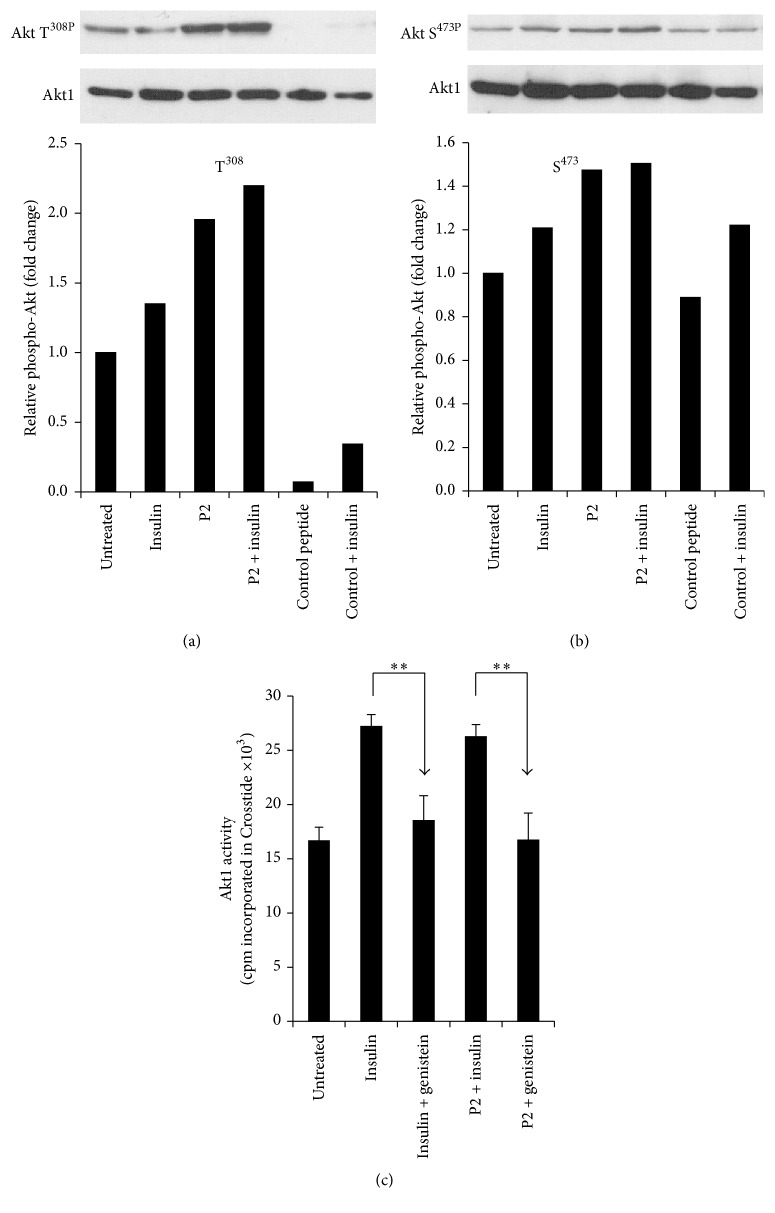
P2 activates Akt. (a) P2 stimulates phosphorylation of Akt T^308^ in cells. HEK cells were serum-starved overnight and treated with the indicated myristoylated peptides (40 *μ*M) for 15 min; insulin (100 nM) was added for the final 10 min of treatment. Cell lysates were analyzed by Western blot; the blot was probed with antibody to phospho-Akt T^308^, followed by anti-Akt1 to control for loading. Signals were quantified by densitometry, and the phospho-Akt : total Akt1 ratio is expressed relative to that of untreated cells (control peptide; KKEVGKD). (b) P2 stimulates phosphorylation of Akt S^473^ in cells. A Western blot of identical lysates to those in (a) was probed with antibody to phospho-Akt S^473^, followed by anti-Akt1. Quantification is as in (a). (c) P2-mediated activation of IRK resulted in stimulation of Akt kinase activity in cells. Cells were serum-starved overnight and treated with myristoylated peptides and insulin as in (a). Genistein (50 *μ*M) was added to cells 30 min prior to treatment with peptide or insulin. Akt1 was immunoprecipitated from the lysates and kinase activity was measured using Crosstide as substrate ^*∗∗*^ *P* < 0.01.

Activation of IRK leads to activation of PI3K, which in turn activates PDK1 followed by recruitment of Akt to the cell membrane. Activated PDK1 phosphorylates Akt at T^308^, thereby activating the kinase, after which maximum activation is achieved by phosphorylation at S^473^. We examined the phosphorylation status of these residues in cells that had been treated with myristoylated P2, insulin, or both, by Western blotting. An additional control was control peptide (KKEVVGKD), which has only distant similarity to P2 (Section 2), and lacks crucial residues for activity [48]. The blots were analyzed by densitometry to quantify the extent of phosphorylation. Peptide treatment resulted in enhanced phosphorylation at both sites (Figures 5(a) and 5(b)). Akt kinase activity was then measured in cells loaded with either P2 or the scrambled P2 peptide, P2sc. Cell lysates were immunoprecipitated with anti-Akt antibody and immunoprecipitates were examined for phosphorylation of Crosstide, an Akt specific substrate. The P2-treated cells displayed increased kinase activity compared to untreated or P2sc controls (Figure 5(c)); the response to P2 was indistinguishable from that to insulin. Activation of Akt in this experiment is almost certainly a consequence of activation of IRK, since the tyrosine kinase inhibitor genistein prevented Akt activation (Figure 5(c)) by either insulin or P2.

### 3.6. P2 Treatment of Cells Inactivates GSK3

**Figure 6:**
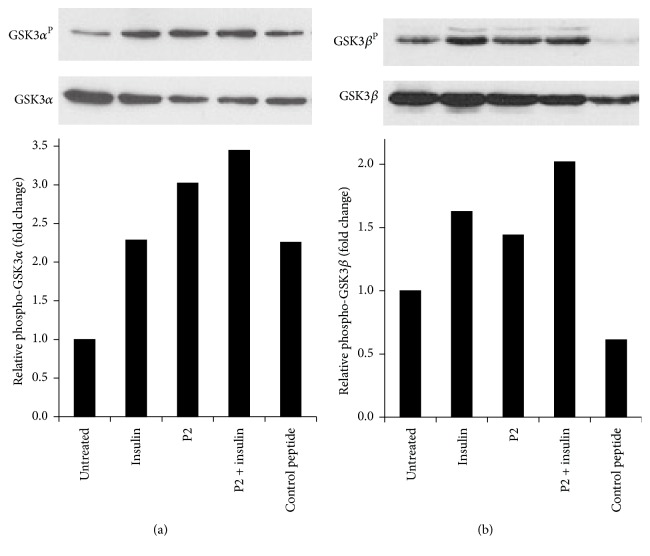
P2 inactivates GSK3. (a) Phosphorylation of GSK3*α* in P2-stimulated cells. Lysates of cells treated as in Figure 5(a) were analyzed by Western blot; the blot was probed sequentially with antibodies to phospho-GSK3*α*-S^21^ and GSK3*α*. The signals were quantified by densitometry as in Figure 5. (b) Phosphorylation of GSK3*β* in P2-stimulated cells. Identical samples to those in (a) were probed with anti-phospho-GSK3*β*-S^9^ antibody, followed by anti-GSK3*β*; signals were quantified as in (a).

Glucose taken up by cells is either metabolized or converted to glycogen for storage. Which pathway is chosen is dependent on the activity of glycogen synthase, which is inversely regulated by its phosphorylation. Namely, both GSK3*α* phosphorylated at S^21^ and GSK3*β* phosphorylated at S^9^ by Akt are inactive, resulting in activation of glycogen synthase and hence increased glycogen synthesis. We therefore examined the phosphorylation status of each isoform in cells treated with peptides or insulin, using the same regimen as that described for Akt phosphorylation. As expected, both GSK3 isoforms displayed enhanced phosphorylation in response to insulin or to myr-P2 (Figure 6), as indicated by Western blotting and densitometry. Thus, the consequences of activation of IRK or IGFRK by P2 manifest themselves throughout the IRK signaling pathway and closely resemble the effects of insulin or IGF1.

## 4. Discussion

We have described in this report a novel method of delivering an hBVR-based peptide to intact animals and its efficacy in ameliorating hyperglycemia* in vivo*. In our previous studies, we had used N-myristoylated peptides for this purpose; these were readily taken up by cultured cells and were shown to activate or inhibit target kinases in the same manner as was observed* in vitro* [16, 38, 48, 54]. In particular, the peptide P2 (KYCCSRK) was shown to stimulate glucose uptake by cultured cells [48]. Despite the successful use of the myristoylated peptides, we were aware that they were probably not stable in the cell and that they were unlikely to be of use* in vivo*, due to their small size, which makes them susceptible to rapid excretion via the kidney. Recently, we described a DNA-based method of peptide delivery to cells: a plasmid that expressed a fusion protein with the peptide sequence immediately following a spontaneously cleaved linker [48, 55, 56]. The expression plasmid was delivered to cell by transfection, and increased uptake of glucose by the transfected cells was shown to persist for up to 48 h. The stability of the peptide is unlikely to have been improved by this approach; however, continuous synthesis driven by a highly active promoter shifts the stability issue back to the retention of plasmid by the cells. Moreover, it is difficult to quantify the level of peptide in the cell at steady state, so that controlling the dose of peptide is problematic. The system described here overcomes these problems. Using a peptide with blocked N- and C-termini, as well as D-amino acids, overcomes the difficulties with peptide stability. Cultured cells treated with nanoparticles for 1 h demonstrated a 125% increase in glucose uptake and a 90% increase after 6 h (Figure 2(b)). In contrast, cells treated with myristoylated peptide showed no significant change in glucose after 6 h. This increased glucose uptake in the presence of nanoparticles was dose-dependent and to a large extent independent of the cell type used.

Previously, we had demonstrated the efficacy of P2 in activating IRK [48] and had noted that the K^1^, K^7^, and C^3^ were each necessary for the peptide to activate the kinase, while replacement of C^4^ resulted in less than full activity. Similarly, replacement of Y^2^ with F led to reduced activation of IRK (Figure 4(c)). Other peptides that included the IRK phosphorylation targets Y^198^ and Y^228^ were phosphorylated by IRK* in vitro*; neither peptide stimulated glucose uptake, and, in one case, the peptide bound avidly to the active site and effectively inhibited kinase activity [48]. We examined other BVR-based peptides in this study. While none of those tested affected autophosphorylation of IRK, they were, to varying degrees, inhibitors of* in vitro* phosphorylation of insulin receptor substrate peptide. In the case of the peptide P1, this was also true in cultured cells treated with insulin or IGF1.

**Figure 7:**
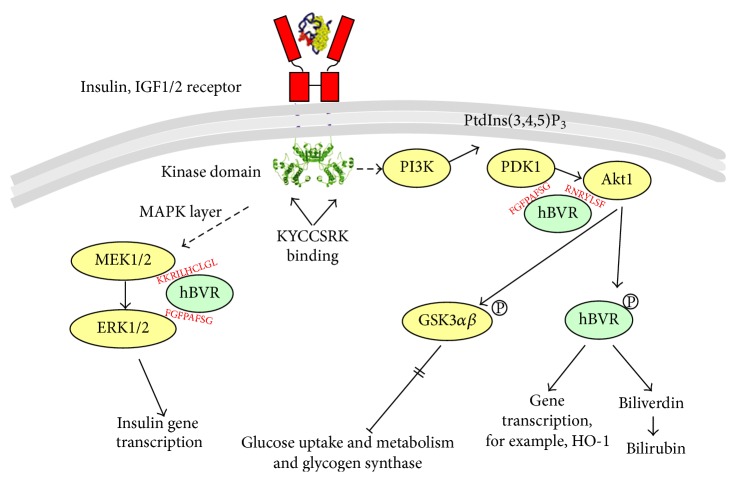
P2-dependent activation of the IRK/PI3K/Akt pathway. IRK activates both MEK/MAPK and PI3K/Akt pathways. On one side, activated PI3K [13, 14] generates PtdIns(3,4, 5)P_3_, which interacts with the PH domain of Akt and recruits the inactive protein to the membrane, where it is phosphorylated at T^308^ by the 3-phosphoinositide-dependent kinase, PDK1 [15]. On the other side, BVR interacts with MEK1/2 and ERK1/2 to enhance activation of ERKs [16]. Activation of ERK1/2 results in activation of transcription factors to stimulate expression of the insulin gene [17]. The peptide, P2 (KYCCSRK), binds to the receptor kinase domain, which results in activation of the multistep process leading to activation of MEK1/2 in the MAPK pathway. Specific sequence motifs in hBVR mediate its interactions with proteins in the signaling pathways, where it acts as a scaffold to bring kinases in contact with their substrates [16]. Akts phosphorylate GSK3*α*, *β* resulting in its inactivation; the inactive GSK3s in turn cannot phosphorylate and inactivate glycogen synthase, resulting in an activation of glucose uptake.

In our previous studies with both the intact hBVR protein and P2, we focused on two narrow aspects of the insulin receptor response: activity of the receptor kinase, which is clearly at the start of signaling, and glucose uptake, which is the end result of activation of one of the many pathways that respond to activated IRK [12, 48]. In this study, we examined the effect of P2 on two intermediate steps in the insulin response, as summarized in Figure 7. An early step in the insulin response pathway is activation of PI3K, which in turn results in activation of PDK1 and recruitment of Akt to the membrane. PDK1 phosphorylates T^308^ in the Akt activation loop. The partially activated kinase is further phosphorylated at S^473^ by several different kinases. T^450^ is also phosphorylated, but this has little or no effect on the kinase activity. We noted in Figures 5(a) and 5(b) that phosphorylation of both of these sites is increased in response to P2. As expected, this results in an increased activity of the kinase (Figure 5(c)). The activation of Akt is also reflected in the phosphorylation of the GSK3*α* and GSK3*β* isozymes (Figure 6). These proteins are inactivated by phosphorylation, leading to increased activity of their downstream targets, notably glycogen synthase [36, 57], which is a key regulatory step in glycogen synthesis and glycolysis. GSK3 is also a regulator of transcription factors, including NRF2 and the AP1 subunits cFos and cJun (Figure 7) [58–60].

Complications of type 2 diabetes are related to oxidative stress as obesity and insulin resistance have predominant role in it [61, 62]. In addition to its direct interaction with the kinase domain of the insulin receptor, KYCCSRK indirectly mitigates untoward effects of hyperglycemia by regulating cellular levels of the heme oxygenase isozymes, HO-1 and HO-2 [63]. Peptide treatment of cells stabilizes BVR mRNA and, in turn, hBVR activation stabilizes HO-2 mRNA and protein [64]. HO-2 is a redox-sensitive K^+^/Ca^2+^ channel-associated protein [65]. BVR is also essential for transcriptional activation of HO-1 by AP1/2-regulated genes, c-jun and CREB/ATF-2 [35, 36, 42]. Both HO isozymes are the sole source of CO and bilirubin, which are essential components of the cellular defense mechanisms. The bile pigments protect against inflammation, xenograft rejection, vasoconstriction, and oxidative stress [32, 66–74]. The protective functions described are in part offset by the observation that HO-dependent formation of CO in the brain leads to inhibition of neuropeptide release, notably arginine vasopressin and corticotrophin releasing hormone [75–77]. This in turn exacerbates the systemic inflammatory response. Expression of HO-1 improves glucose metabolism [78]. HO-2 deficiency in HO-2^(−/−)^ mice has been reported to contribute to diabetes-mediated increase in superoxide anion and renal dysfunction [79]. KYCCSRK also stimulates BVR reductase activity that is essential for conversion of biliverdin, the immediate product of HO-1 and HO-2 activity, to bilirubin [38].

P2 delivered in nanoparticles was shown to be effective in reducing blood glucose in intact animals, when compared with a control peptide with the same amino acid composition but entirely different sequence. Circulating glucose in wt mice that had been pretreated with peptide and then injected with glucose was decreased compared to controls in this glucose tolerance test (Figure 3(a)). Similarly, genetically obese mice also showed decreased blood glucose in response to P2 treatment and were able to better tolerate the hyperglycemia that arose from ketamine-xylazine anesthesia (Figures 3(b) and 3(c)). Further, treatment with P2-nanoparticles prevented glucose stimulation of insulin secretion, indicating that peptide-dependent stimulation of glucose uptake and hence lowering of circulating glucose were bypassing the physiological need for insulin.

## 5. Conclusions

The data obtained in this study point to a new method for controlling hyperglycemia that should be efficacious in both type 1 and type 2 diabetes. By targeting the insulin receptor kinase directly and thereby activating the full range of downstream pathways radiating from the insulin receptor, the peptide may prove to be an alternative or addition to other drugs currently used for reducing hyperglycemia. We have recently examined the interaction of the peptide with IRK and have demonstrated that the peptide induces a conformational change in the kinase domain of the receptor [48] as does insulin. This analysis enabled determination of peptide's binding site to the kinase domain and the conformation of the protein. It is reasonable to suggest that further structural analyses would enable design of modified peptide that would be more efficient in increasing cellular glucose uptake than the native peptide.

## Abbreviations

hBVR: Human biliverdin reductase
IRK: Insulin receptor kinase
Akt1/PKB: Protein kinase B
GSK3: Glycogen synthesis kinase 3
HEK cells: Human embryonal kidney cells
HSKM: Human skeletal muscle cells
IGF1: Growth factor 1.
